# Cetuximab combined with paclitaxel or paclitaxel alone for patients with recurrent or metastatic head and neck squamous cell carcinoma progressing after EXTREME

**DOI:** 10.1002/cam4.3953

**Published:** 2021-05-25

**Authors:** Thomas Chevalier, Amaury Daste, Esmaa Saada‐Bouzid, Anderson Loundou, Florent Peyraud, Tiphaine Lambert, Christophe Le Tourneau, Frédéric Peyrade, Charlotte Dupuis, Marc Alfonsi, Jérôme Fayette, Juliette Reure, Florence Huguet, Nicolas Fakhry, Clémence Toullec, Sébastien Salas

**Affiliations:** ^1^ Department of Medical Oncology CHU la Timone AP‐HM Marseille France; ^2^ Department of Medical Oncology Hôpital Saint‐André Bordeaux University Hospital‐CHU Bordeaux France; ^3^ Department of Medical Oncology Centre Antoine Lacassagne Nice France; ^4^ EA3279 Self‐Perceived Health Assessment Research Unit Aix‐Marseille University Marseille France; ^5^ Department of Drug Development and Innovation (D3i) Paris‐Saclay University Institut Curie Paris & Saint‐Cloud France; ^6^ Department of Radiation Oncology Clinique Sainte Catherine Avignon France; ^7^ Department of Medical Oncology Léon Bérard Center University of Lyon Lyon France; ^8^ Department of Radiation Oncology Tenon Hospital Paris Sorbonne Université, Assistance Publique‐Hôpitaux de Paris Paris France; ^9^ Department of Otorhinolaryngology ‐ Head and Neck Surgery AP‐HM Aix‐Marseille University France; ^10^ Department of Medical Oncology Clinique Sainte Catherine Avignon France

**Keywords:** cetuximab, chemotherapy, EXTREME, paclitaxel, Recurrent/metastatic head and neck squamous cell carcinoma

## Abstract

**BACKGROUND:**

Prognosis of recurrent or metastatic (R/M) head and neck squamous cell carcinoma (HNSCC) remains poor. The addition of cetuximab, to platinum and fluorouracil chemotherapy (EXTREME regimen) has been shown to improve patients’ outcomes in first‐line settings.

**METHODS:**

We conducted a retrospective, multicenter study, including HNSCC that progressed after a first line of platinum‐based chemotherapy and cetuximab, treated either by paclitaxel + cetuximab (PC) or paclitaxel alone (P), between January 2010 and April 2018. The end points were overall survival (OS), progression‐free survival (PFS), and overall response rates (ORR). Patients were matched according to their propensity scores, estimated with a logistic regression model. The secondary objectives were to study the safety profile and to look for prognostic and predictive factors of effectiveness.

**RESULTS:**

Of the 340 identified patients, 262 were included in the analysis, 165 received PC, and 97 received P. In unmatched population, ORR was 16.4% with PC and 6.2% for P. Median PFS was 2.9 months [95% Confidence Interval 2.7–3.0] for PC versus 2.5 months [2.2–2.7] for P, hazard ratio (HR) = 0.770 [0.596–0.996]. Median OS was 5.5 months [4.4–6.9] for PC versus 4.2 months [3.4–4.8] for P, HR = 0.774 [0.590–1.015]. In multivariate analysis, PC was associated with better PFS and OS. These results were consistent in matched‐paired population. Previous cetuximab maintenance for more than 3 months was predictive of better OS with PC.

**CONCLUSION:**

Although the continuation of cetuximab in combination with paclitaxel after EXTREME provides moderate benefit, it could be an interesting option for selected patients.

## Graphical abstract

In this propensity score‐adjusted, multi‐institutional series, cetuximab in association with paclitaxel showed better outcomes than paclitaxel alone for patients who had disease progression after EXTREME regimen, particularly for patients who benefit the most from cetuximab in first‐line setting. This study should raise the question of the place of this association with the arrival of immune checkpoint inhibitors in first line with or without chemotherapy.

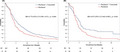

## 1 INTRODUCTION

Head and neck squamous cell carcinoma (HNSCC) is the fifth most frequent and the sixth most common cause of death by cancer. Most patients are diagnosed at a locally advanced stage. Despite progress in primary treatment by combining surgery, radiation therapy, chemotherapy, and supportive care, the recurrence rate is about 40% for all stages.^1^ Prognosis remains poor for patients who are ineligible for salvage therapy. Cetuximab, an epidermal growth factor receptor (EGFR)‐targeting monoclonal antibody, was the first targeted therapy to show a significant benefit in HNSCC. In a single‐agent trial, it showed 13% overall response rate (ORR) and a median time to progression of 70 days in patients with R/M HNSCC who failed to respond to platinum‐based therapy.^2^ Cetuximab combined with platinum‐based chemotherapy followed by cetuximab maintenance (EXTREME) improved progression‐free survival (PFS), overall survival (OS), and ORR compared to platinum‐based chemotherapy alone as the first‐line therapy.^3^

Weekly paclitaxel (P) monotherapy was evaluated in a non‐randomized phase II and showed an objective response in 43.3% of patients with 5.2 months median OS in a first‐line platinum‐refractory setting or as a second line after platinum‐based chemotherapy.^4^ One retrospective study showed similar results,^5^ so it is an option for platin‐resistant R/M HNSCC.^6^ Taxanes and Cetuximab have been shown to have synergistic activity in in vitro studies.^7^ Two non‐randomized phase II trials have evaluated paclitaxel and cetuximab (PC) as first‐line treatment. They showed a 52%–54% response rate and 4.2–7.0 median PFS and 8.1–16.3 median OS.^8^, ^9^ In retrospective studies, PC showed high activity with a 48%–55% response rate, and a median OS of 7.6–9.2 months. However, in these retrospective studies, a significant proportion of patients were treated in a first‐line setting because of their ineligibility for platinum chemotherapy.^10^, ^11^ To the best of our knowledge, continuation of cetuximab after platinum‐based chemotherapy in combination with taxanes has not yet been evaluated.

We conducted a multicenter retrospective study to evaluate the value of continuing cetuximab in association with P beyond progression after an EXTREME chemotherapy regimen +/‐ cetuximab as maintenance in R/M HNSCC. Secondary objectives were to identify the prognostic factors and predictive factors for response to the PC combination and evaluate safety.

## 2 MATERIALS AND METHODS

### 2.1 Study design and data source

We retrospectively collected the medical files of all patients treated with paclitaxel +/‐ cetuximab in seven French centers between January 2010 and April 2018. Patients who met the following criteria were included: histologically confirmed R/M HNSCC, were not eligible for salvage therapy (i.e., radiation therapy and/or surgery) according to local multidisciplinary concertation and had disease progression after first‐line chemotherapy containing platinum and cetuximab +/‐ fluorouracil and +/‐ cetuximab in maintenance. Patients were excluded if they had naso‐sinusal, cutaneous, or parotidean carcinomas, if they had received taxane‐based chemotherapy in a first‐line setting or if they had received more than one previous line of treatment. Patients could have received docetaxel in a neoadjuvant or induction chemotherapy regimen.

We analyzed the following parameters: gender; age; location of primary tumor; initial TNM classification (7^th^ UICC edition); p16 status if available; treatment for localized disease, that is, induction chemotherapy, surgery, chemo radiotherapy (RT‐CT); site of recurrence (locoregional vs. metastatic); first‐line chemotherapy regimen; time to progression after first‐line chemotherapy (TTP1) defined as the time between first injection of platinum +cetuximab +/‐ fluorouracil and clinical or radiological progression; and best response to first‐line chemotherapy according to RECIST 1.1^12^ and World Health Organization (WHO) Performance Status (PS). We considered that patients received cetuximab maintenance if they received an injection more than 21 days after the last platinum injection and had a stable or objective response to the EXTREME regimen. Duration of cetuximab maintenance was defined as the time between the beginning of maintenance and the last infusion of cetuximab. Chemotherapy‐free interval (CFI) was defined as the time between last infusion of chemotherapy with platinum and progression. The study was authorized by the review board of the Groupe d’Oncologie et Radiothérapie Tête et Cou (GORTEC).

### 2.2 Statistical analysis

Characteristics of patients who received PC were compared with those who received P alone using Chi2 or Fisher's exact tests for categorical variables, and the Wilcoxon test for continuous variables.

The objectives were to assess PFS defined as the time from first injection of PC or P to disease progression, assessed clinically or radiologically, or death, OS defined as the time from first injection of PC or P to death, and overall response rate, assessed by CT scan and/or MRI when CT scan was not sufficient to evaluate response according to RECIST 1.1. Patients without events were censored at the time of last follow‐up. Survival curves were generated using the Kaplan–Meier method and compared using the log‐rank test. Hazard ratio (HR) estimations are provided along with their bilateral confidence intervals. A propensity score for receiving PC was estimated using a logistic regression. Patients who received PC were matched on this score to patients who received P alone. The impact of cetuximab on PFS and OS was assessed on this matched population by log‐rank tests stratified on the pairs.

The prognostic impact of the different clinical factors was tested in univariate analysis. Factors that showed individual prognostic value in univariate models with a *p* value of less than 0.2 were used to examine their joint prognostic value in a multivariate model. Patients with missing data were not included in the multivariate analysis for prognostic factors and predictive factors. Tests to determine interactions between treatment and covariates were used in the Cox model to identify predictive factors by assessing whether there was a significant difference in the treatment effect on OS and PFS. Patients were categorized as responders if they had an objective response (i.e., partial or complete response) and non‐responders if they had stable disease or progression. Predictive factors for response to PC were assessed using a logistic regression.

The level of statistical significance was set at α = 0.05. All *p* values are two‐sided. Statistical analyses were carried out with SPS software and Addinsoft (2019) XLSTAT statistical and data analysis solution, version 10.13, Paris France https://www.xlstat.com.

## 3 RESULTS

### 3.1 Characteristics of population

**FIGURE 1:**
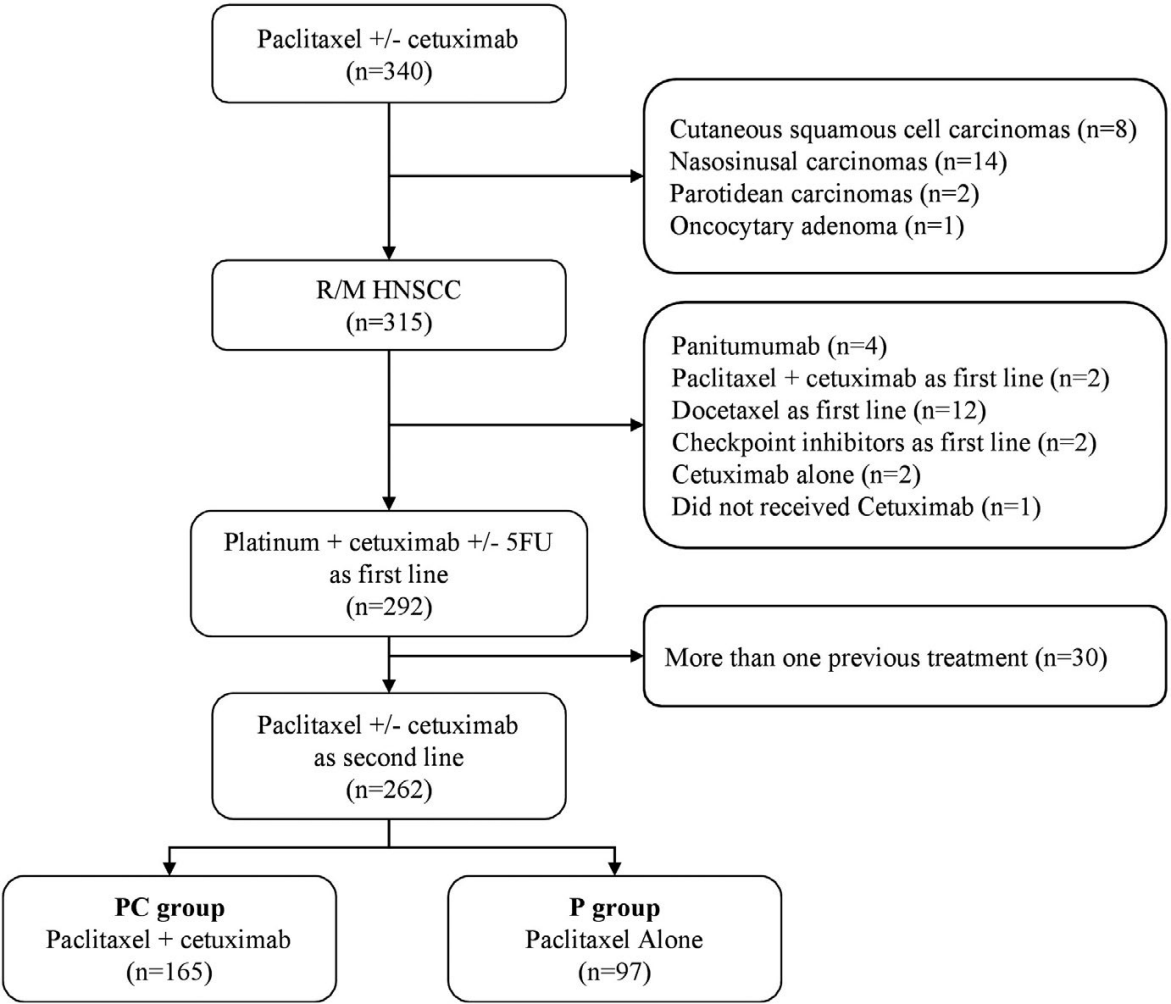
Patient flow diagram showing selection of patients with R/M HNSCC who received Paclitaxel +/‐ Cetuximab as second line. Abbreviations: HNSCC, Head and Neck Squamous Cell Carcinoma, R/M, Recurrent or Metastatic, 5FU, Fluorouracil; PC, Paclitaxel + Cetuximab; P, Paclitaxel

Of 340 patients who received paclitaxel +/‐ cetuximab, a total of 315 patients with R/M HNSCC were identified, including 262 who received EXTREME. Thirty patients were excluded because they had received more than one previous line of treatment. Of the 262 patients who received paclitaxel +/‐ cetuximab, 165 (63%) received PC, and 97 (37%) received P alone (Figure 1). Patients received either P 60 to 80 mg/m^2^ per week, 3 weeks/4, and weekly cetuximab 250 mg/m^2^, or P alone as described above.

As of September 2018, the median follow‐up of patients was 42.1 months (CI 95% 19.1‐NR). PS was not available for 26 patients, 11 in PC, and 15 in P. PS was 0–1 or 2 for the 236 other patients. Ninety percent of patients who received paclitaxel + cetuximab or paclitaxel alone had progressed within 1 month after the last cetuximab injection. The main reasons for stopping cetuximab during maintenance was the occurrence of serious adverse event, or patient's refusal. All of these patients had achieved disease control at the time of the interruption. Baseline characteristics differed by subgroup (Table 1).

**TABLE 1:** Characteristics of patients at baseline in the whole population and in the matched‐paired population

	Unmatched population						Matched‐paired population			
	PC (n = 165)		P (n = 97)		*p value*	PC (n = 70)		P (n = 70)		*p value*
	No.	%	No.	%		No.	**%**	No.	**%**	
Gender										
Male	136	82%	85	88%	0.263	57	81%	59	84%	0.654
Female	29	18%	12	12%		13	19%	11	16%	
Age										
Median [min–max]	61.5 [40–80]		61.7 [34–86]		0.151	62.0 [42–85]		62.2 [34–86]		0.065
<65 years	98	59%	63	65%	0.372	39	56%	46	66%	0.226
≥65 years	67	41%	34	35%		31	44%	24	34%	
Site										
AP‐HM––Marseille	116	70%	5	5%	**<0.0001**	40	57%	4	6%	**<0.0001**
CHU Bordeaux	6	4%	49	51%		5	7%	38	54%	
ISC––Avignon	22	13%	15	15%		21	30%	14	20%	
CAL––Nice	20	12%	12	12%		3	4%	12	17%	
Curie––Paris	0	0%	12	12%		0	0%	0	0%	
Tenon	1	1%	2	2%		1	1%	2	3%	
CLB––Lyon	0	0%	2	2%		0	0%	0	0%	
Location										
Oral cavity	43	25%	21	21%	0.422	12	17%	15	21%	0.520
Hypopharynx	37	22%	14	14%	0.057	10	14%	10	14%	1.000
Larynx	29	18%	10	10%	0.111	8	11%	6	9%	0.573
Oropharynx	52	31%	50	51%	**<0.001**	38	54%	35	50%	0.612
Unknown Primary	8	5%	4	4%	0.786	2	3%	4	6%	0.404
Initial TNM classification (7th UICC)										
Stage I	7	4%	7	7%	0.189	2	3%	5	7%	0.145
Stage II	23	14%	6	6%		8	11%	4	6%	
Stage III	25	16%	18	19%		11	16%	12	17%	
Stage IV	105	66%	66	68%		45	64%	49	70%	
P16 Status										
Positive	5	3%	3	3%	0.110	3	4%	2	3%	**<0.0001**
Negative	25	15%	57	59%		13	19%	41	59%	
Missing	135	82%	37	38%		54	77%	27	39%	
Primary treatment										
Induction CT	42	25%	14	14%	**0.036**	19	27%	11	16%	0.099
Surgery	57	33%	38	39%	0.452	21	30%	29	41%	0.158
RT+/‐CT	149	90%	70	72%	**<0.001**	57	81%	54	77%	0.532
Site of recurrence										
Locoregional	79	48%	41	43%	0.419	35	50%	31	44%	0.505
Metastatic	86	52%	55	57%		35	50%	38	54%	
First‐line chemotherapy										
Cisplatin	69	42%	41	42%	0.943	27	39%	32	46%	0.392
Carboplatin	125	76%	77	79%	0.500	53	76%	55	79%	0.687
Fluorouracil	130	79%	64	66%	**0.022**	55	79%	51	73%	0.430
Response to EXTREME										
Yes	64	39%	35	36%	0.664	27	39%	30	43%	0.606
No	101	61%	62	64%		43	61%	40	57%	
TTP1										
<6 months	88	53%	54	56%	0.714	40	57%	38	54%	0.734
≥6 months	77	47%	43	44%		30	43%	32	46%	
Chemo‐free Interval										
<3 months	104	63%	65	67%	0.516	45	64%	43	61%	0.726
≥3 months	61	37%	32	33%		25	36%	27	39%	
Cetuximab maintenance										
No maintenance	84	51%	66	68%	**0.025**	41	59%	42	60%	0.910
<3 months	43	26%	17	18%		14	20%	15	21%	
≥3 months	38	23%	14	14%		15	21%	13	19%	
Performance Status										
0–1	106	64%	45	46%	**0.008**	41	59%	39	56%	0.733
2	48	29%	37	38%		29	41%	31	44%	
Missing	11	7%	15	16%						

Note

Chi2 was used to generate *p* values.

Bold values indicate values that are statistically different.

Abbreviations: CT, Chemotherapy; No, Number, NS, Non significative; P, Paclitaxel;PC, Paclitaxel +cetuximab; RT, Radiation Therapy; TTP1, Time to progression under EXTREME first‐line chemotherapy; UICC, Union for International Cancer Control.

### 3.2 Unmatched population

**FIGURE 2:**
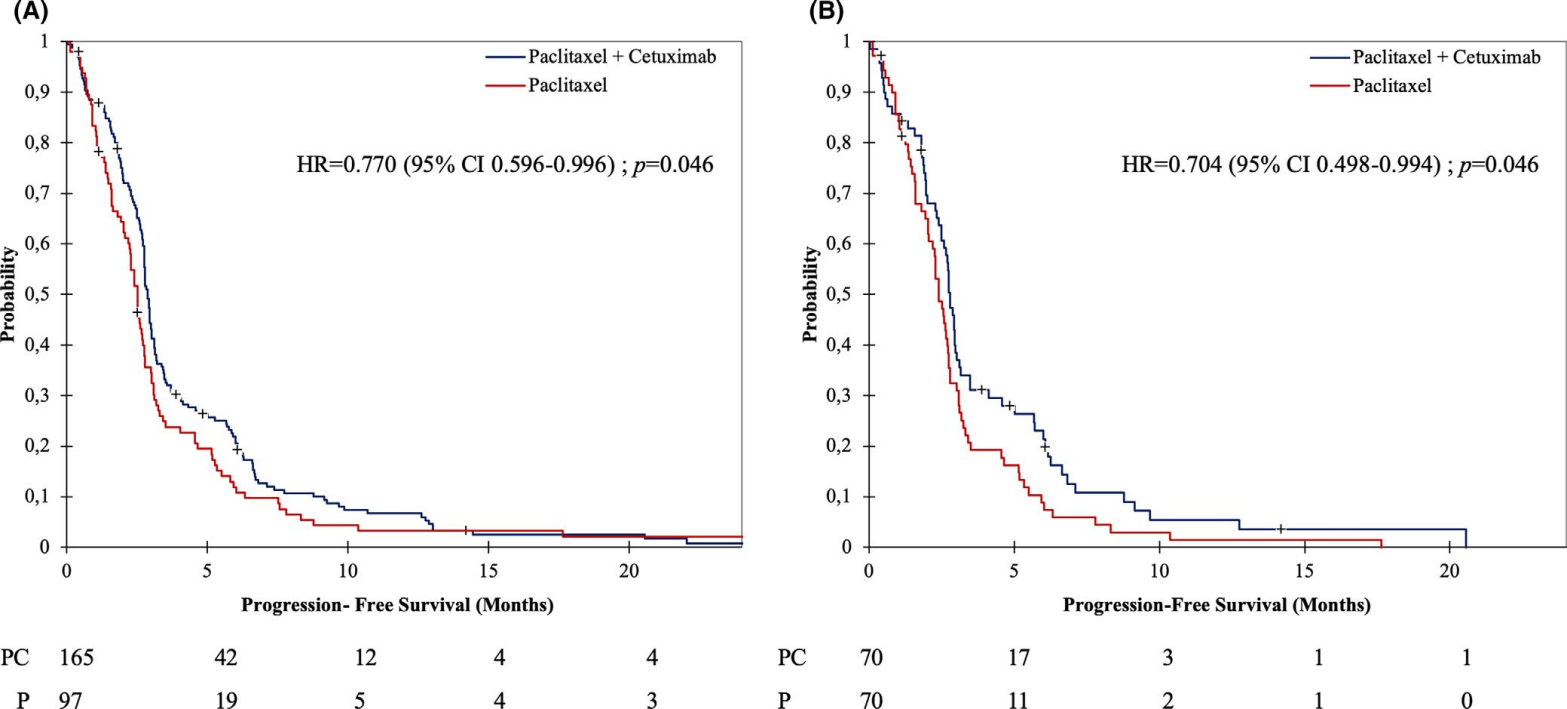
Kaplan–Meier for PFS in overall population (A) and in matched‐paired population (B). *p* value calculated with log‐rank test. Abbreviations: HR, Hazard Ratio; CI, Confidence interval

**FIGURE 3:**
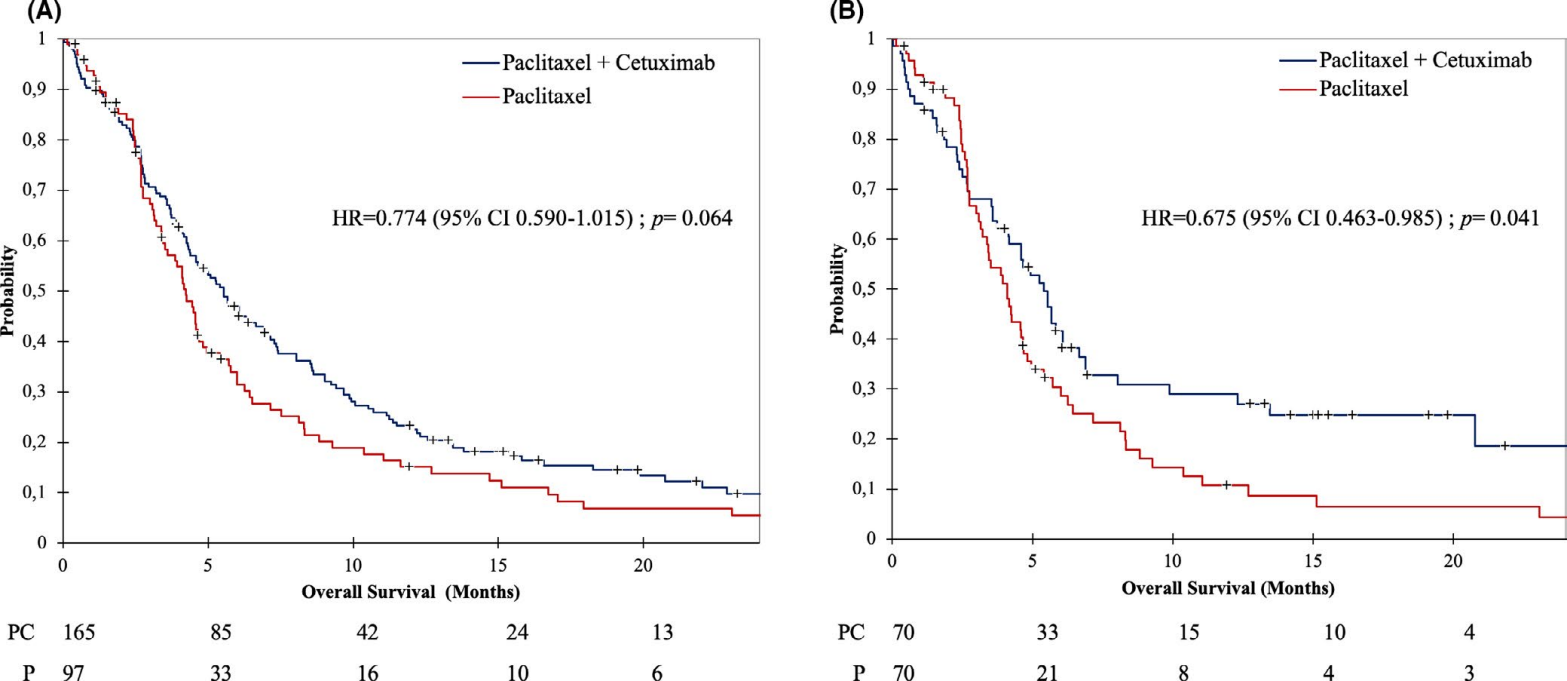
Kaplan–Meier for OS in overall population (A) and in matched‐paired population (B). *p* value calculated with log‐rank test. Abbreviations: HR, Hazard Ratio; CI, Confidence interval

A total of 253 PFS events (96.6%, 159/165 and 94/97 in patients treated with PC and P, respectively), were observed. Unadjusted median PFS was 2.9 months [95% CI 2.8–3.0] and 2.5 months [95% CI 2.2–2.7], in PC and P, respectively, HR= 0.770 [95% CI 0.596–0.996]; *p* = 0.046 (Figure 2). Death occurred in 230 of 262 patients (87.8%), 145/165 (87.9%) in the PC group versus 85/97 (87.6%), in the P group. Unadjusted median OS was 5.5 months [95% CI 4.4–6.9] in the PC group versus 4.2 months [95% CI 3.4–4.8] in the P group. OS was not significantly longer in the PC group in univariate analysis, HR = 0.774 [95% CI 0.590–1.015]; *p* = 0.064 (Figure 3). In the PC group, 27 of 165 patients (16.4%) achieved an objective response versus 6 of 97 (6.2%), in the P group (OR = 2.97 [95% CI 1.18–7.47]; *p*=0.021). Two patients in the PC group had complete response. However, disease control rates (i.e., objective response and stable disease) did not significantly differ between the two groups with 32% and 24% in the PC and P groups, respectively (*p* = 0.147). Median duration of response was 7.7 months [95% CI 6.7–12.8] for PC and 5.5 months [95% CI 5.1–17.6] for P alone (log‐rank test, *p* = 0.391).

Adjusting for the baseline factors (tumor location, cetuximab maintenance, CFI, TTP1, WHO PS, second‐line chemotherapy, and response to EXTREME for OS or age for PFS) in multivariate analysis, revealed a significant difference in terms of PFS (HR = 0.708, 95% CI 0.529–0.948, *p* = 0.021) and OS (HR = 0.712, 95% CI 0.519–0.980, *p* = 0.037) for PC. In this multivariate analysis, TTP1 ≥ 6 months was associated with better OS (*p* = 0.032) and WHO PS with better PFS and OS (*p* = 0.001 and *p* < 0.0001, respectively) (Table 2).

**TABLE 2:** Multivariate analysis for Overall Survival (OS) and Progression‐Free Survival (PFS) using Cox model including location of primary, Time to progression under EXTREME first‐line chemotherapy (TTP1), cetuximab maintenance, second‐line chemotherapy, World Health Organization Performance Status (WHO PS), age for PFS, objective response to EXTREME for OS

	PFS		OS	
	HR [95% CI] UV / MV	*p* value UV / MV	HR [95% CI] UV / MV	*p* value UV / MV
Paclitaxel +Cetuximab versus Paclitaxel	0.77 [0.60–0.99] / 0.71 [0.53–0.95]	**0.046 / 0.021**	0.77 [0.59–1.01] / 0.71 [0.52–0.98]	0.064 / **0.037**
Male versus Female	1.14 [0.81–1.60] / NP	0.460 / NP	0.89 [0.61–1.28] / NP	0.520 / NP
P16+ versus P16‐	0.74 [0.32–1.74] / NP	0.497 / NP	0.81 [0.35–1.91] / NP	0.638 / NP
Induction chemotherapy Yes versus No	1.08 [0.80–1.46] / NP	0.611 / NP	1.01 [0.73–1.39] / NP	0.958 / NP
Surgery Yes versus No	1.05 [0.81–1.36] / NP	0.701 / NP	1.07 [0.82–1.40] / NP	0.623 / NP
RCT Yes versus No	1.12 [0.66–1.28] / NP	0.606 / NP	0.92 [0.64–1.33] / NP	0.676 / NP
Age ≥65 years versus <65 years	0.81 [0.63–1.05] / 0.78 [0.59–1.03]	0.112 / 0.084	1.04 [0.80–1.37] / NP	0.750 / NP
Localization				
Oral cavity	1.21 [0.91–1.62] / 1.62 [0.81–3.23]	0.191 / 0.173	1.35 [0.98–1.80] / 1.28 [0.64–2.57]	0.070 / 0.491
Hypopharynx	0.63 [0.46–0.86] / 1.02 [0.50–2.01]	**0.004** / 0.959	0.67 [0.48–0.93] / 0.74 [0.36–1.54]	**0.018** / 0.418
Larynx	0.85 [0.60–1.20] / 1.20 [0.57–2.52]	0.348 / 0.627	0.89 [0.61–1.28] / 0.94 [0.44–2.02]	0.523 / 0.872
Oropharynx	1.49 [1.15–1.93] / 1.65 [0.83–3.29]	**0.003** / 0.153	1.18 [0.89–1.55] / 1.01 [0.51–2.01]	0.239 / 0.975
Unknown Primary	0.71 [0.38–1.34] / NP	0.292 / NP	1.01 [0.54–1.91] / NP	0.972 / NP
Locoregional versus Metastatic	1.13 [0.88–1.44] / NP	0.350 / NP	1.17 [0.90–1.52] / 1.12 [0.84–1.51]	0.244 / NP
TTP1 < 6 months versus ≥6 months	1.46 [1.14–1.88] / 1.47 [0.99–2.20]	**0.003 /** 0.058	1.63 [1.25–2.13] / 1.60 [1.04–2.46]	**0.0003 / 0.032**
EXTREME OR versus Non‐OR	0.87 [0.67–1.30] / NP	0.298 / NP	0.76 [0.58–1.00] / 0.95 [0.66–1.35]	**0.047** / 0.759
Maintenance Yes versus No	0.70 [0.55–0.91] / 1.01 [0.66–1.54]	**0.008** / 0.957	0.80 [0.61–1.04] / 0.84 [0.54–1.29]	**0.004** / 0.418
CFI <3 months versus ≥3 months	1.31 [1.01–1.70] / 1.11 [0.69–1.79]	**0.043** / 0.680	1.51 [1.14–1.99] / 0.89 [0.54–1.46]	**0.004** / 0.634
Maintenance <3 months versus ≥3 months	1.05 [0.71–1.54] / NP	0.806 / NP	1.00 [0.66–1.50] / NP	0.989 / NP
WHO PS 2 versus 0–1	1.55 [1.18–2.04] / 1.61 [1.21–2.14]	**0.002 / 0.001**	1.89 [1.42–2.51] / 1.89 [1.40–2.55]	**<0.0001 / <0.0001**

Bold values indicate differences that are statistically significant.

Abbreviations: 95% CI, 95% Confidence interval; CFI, Chemotherapy‐Free Interval; HR, Hazard Ratios; MV, Multivariate analysis; NP, Not performed; OR, Objective Response; RCT, Radiation +/‐ Chemotherapy; TTP, Time to Progression with first‐line chemotherapy; UV, Univariate Analysis; WHO PS, World Health Organization Performance status; WHO PS, World Health Organization Performance Status.

### 3.3 Predictive factors

We then searched for clinical predictive factors of PC efficacy in the whole population. There was no significant difference in OS between PC and P alone regarding gender, age, tumor location, site of recurrence, response to EXTREME, cetuximab maintenance, and CFI, *p* > 0.05. Patients who had cetuximab maintenance for more than 3 months (n = 52) were more likely to benefit from the association, HR for OS with PC versus P alone was 0.397 [95% CI 0.204–0.774]; *p* = 0.007, whereas it was 1.126 [95% CI 0.570–2.223]; *p* = 0.733, for patients who had cetuximab maintenance for less than 3 months (n = 60) in PC versus P alone. *p* value for interaction between treatment group and duration of cetuximab maintenance on OS was 0.033. For patients with TTP1 ≥ 6 months and CFI ≥3 months, median OS was higher with PC than P (HR = 0.663 [0.439–1.000] and HR = 0.605 [0.375–0.976]), whereas there was no significant difference for patients with TTP1 < 6 months (HR = 0.937 [0.450–1.130]) or CFI <3 months (HR = 0.932 [0.670–1.296]). *p* value for the interaction test was 0.216 for TTP1 and 0.146 for CFI,. Regarding PFS, a hypopharyngeal localization was less likely to benefit from the cetuximab continuation compared to other locations, HR for progression was 1.833 [0.959–3.506]; *p* = 0.067, in PC versus P alone, *p* value for interaction was 0.002. Patients with WHO PS 0–1 had a better prognosis than PS 2–4 (cf. above), but there was no difference in OS between PC and P alone (*p* = 0.251). In contrast, patients with PS 2–4 had better OS (HR = 0.566 [0.359–0.894], *p* = 0.015) and PFS (HR=0.467, [95% CI 0.298–0.730]; *p* = 0.001) with PC than with P, but *p* values for the interaction were not significant, *p* = 0.260 and *p* = 0.069. All results are summarized in the forest plot (Tables S1 and S2). OS Kaplan–Meier curves for TTP1, WHO PS, duration of cetuximab maintenance and CFI in both groups are shown in Figure S2.

We then looked for predictive factors of response to PC using a logistic regression model. Male sex, age <65 years old, non‐responders to EXTREME, TTP1 < 6 months, and CFI <3 months were more likely to have an objective response with PC than with P alone (*p* < 0.05). However, we did not find any predictive factors of response, as *p* value for the interaction was >0.05.

### 3.4 Propensity score analysis

We used the covariates that were significantly associated with PC use (*p* < 0.05) in multivariate analysis, namely, oropharyngeal localization, RTCT, cetuximab maintenance and PS, to which we added TTP1, for which a prognostic impact was found on survival, to calculate the propensity scores. Site was not taken into account for the calculation of the propensity score because the choice of treatment by PC or P was strongly linked to the habit of the center, neither P16 status, we have chosen not to take into account due to the number of missing data. Patients were then matched on these propensity scores (based on a calliper width of 0.50 of the log odds of the propensity score). The model exhibited acceptable discrimination capability, with an area under the curve equal to 0.7336 (Figure S1) and good calibration, with a *p* value for the Hosmer‐Lemeshow goodness of fit test equal to 0.9199.

### 3.5 Matched‐paired population

After propensity score matching 140 patients with a 1:1 ratio, 70 in each group, baseline characteristics did not differ between PC and P alone groups with the exception of site and P16 status (Table 1). In the 1:1 matched‐paired population, PC showed significantly longer PFS (median, 2.8 months; 95% CI 2.5–3.0) in comparison with P alone (median, 2.4 months; 95% CI 2.1–2.7), HR for progression was 0.704 [95% CI 0.498–0.994], *p* = 0.046 (Figure 2). Six months progression‐free survival rates were 21% (95% CI 11%–31%) and 7% (95% CI 1%–14%) for PC and P, respectively. The estimated median OS was longer with PC (5.4 months; 95% CI 4.1–6.7) than with P alone (4.1 months; 95% CI 3.2–4.7), HR for death was 0.675 [95% CI 0.463–0.984]; *p* = 0.041 (Figure 3). The estimated 1‐year survival was 27% [95% CI 16%–38%] and 9% [95% CI 1%–16%] in the PC and the P groups, respectively.

There was no difference in ORR with 11.4% and 8.6% for PC and P, respectively, OR = 1.376 [95% CI 0.451–4.196]; *p* = 0.576.

### 3.6 Safety

Safety data were available for 148 patients in the PC group and 89 patients in the P group. The main toxicities observed are summarized in Table 3. We found that neutropenia, anemia, and nausea, were more frequent inpatients receiving PC. Hematotoxicity often occurred within the first cycle of treatment, probably because of reminiscent effect of platinum chemotherapy. Specific toxicities of cetuximab such as skin toxicities, hypomagnesemia, or hypocalcemia occurred as expected and was manageable with doxycycline and local treatments. Treatment discontinuation for toxicity was similar in both groups: 9.7% and 12.1% in the PC and P groups, respectively, *p* = 0.672.

**TABLE 3:** Safety

	All Grades		*p* value	Grade 3–5		*p* value
	PC (*n* = 150)	P (*n* = 91)		PC (*n* = 150)	P (*n* = 91)	
Anemia	67%	32%	**<0.0001**	11%	7%	0.287
Neutropenia	37%	15%	**0.0004**	20%	7%	**0.010**
Febrile neutropenia	4%	0%	0.053	4%	0%	0.053
Thrombocytopenia	9%	7%	0.455	0%	0%	
Sensitive neuropathy	35%	34%	0.841	4%	3%	0.780
Asthenia	61%	60%	0.890	0%	0%	
Diarrhea	13%	5%	0.071	0%	0%	
Nausea	21%	3%	**0.0001**	0%	0%	
Increased transaminases	6%	1%	0.064	1%	0%	0.269
Folliculitis	53%	0%	**<0.0001**	5%	0%	**0.025**
Dry skin	33%	1%	**<0.0001**	0%	0%	
Digital cracks	25%	1%	**<0.0001**	0%	0%	
Hypocalcemia	13%	0%	**0.0003**	1%	0%	0.269
Hypomagnesemia	44%	1%	**<0.0001**	7%	0%	**0.008**

Bold values indicate differences that are statistically significant.

Abbreviations: P, Paclitaxel; PC, Paclitaxel + cetuximab.

### 3.7 Subsequent lines

Finally, we sought to study what had been the subsequent treatments received. Forty‐five percent and 28% of patients in the PC group and in the P group received a subsequent therapy (*p* = 0.018). Subsequent lines mainly consisted in monotherapy with methotrexate, vinorelbine, gemcitabine, polychemotherapy rechallenging platinum‐based chemotherapy with cetuximab, targeted therapy such as Pi3 K inhibitors or immune checkpoints inhibitors. Although more patients received subsequent therapy in the PC group, among the patients who did, 15% and 19% received immune checkpoints inhibitors in the PC and P groups, respectively (*p* = 0.637).

## 4 DISCUSSION

We evaluated the outcome of patients with R/M HNSCC who received paclitaxel +/‐ cetuximab after progression under the EXTREME regimen in a large multicenter retrospective cohort. In the matched‐paired population and in multivariate analysis, PC was associated with better PFS and OS than P alone. Our findings should be interpreted within the limitations of the study design. First, the retrospective nature of this investigation inherently introduces selection bias. We attempted to minimize this with propensity score‐based matching. Although propensity matching may be effective in minimizing the impact of observable confounders, it may not address unobservable confounders that could influence survival.

The observed benefit was low with an improvement of 0.4 months and 1.3 months in median PFS and OS. Given the low benefit and the greater occurrence of side effects such as anemia, neutropenia, and cetuximab‐related skin toxicities, it would be interesting to assess the impact on the quality of life of patients. In exploratory analysis, we found that patients who had TTP1 ≥6 months and cetuximab maintenance ≥3 months could benefit from cetuximab continuation in association with P. Duration of maintenance was the only predictive factor of survival for cetuximab maintenance. We hypothesized that patients who received the most cetuximab in first line could also benefit from the maintenance of therapeutic pressure on EGFR. The fact that patients in poor general condition may benefit more from the association may be partly explained by the 2.6‐fold higher response rate. Toxicity was as expected and manageable.

To the best of our knowledge, this is the largest cohort evaluating PC and the first to investigate whether the cetuximab maintenance beyond the first line in HNSCC.

Median PFS, OS, and response rates were significantly lower than in studies that have already studied this association.^8^, ^9^, ^10^, ^11^ However, in the publications of Enokida et al. and Hitt et al., all patients were treated in a first‐line setting and no patient had already been exposed to cetuximab,^8^, ^9^ which could explain the difference. In the two retrospective studies published by Péron and Fayette, patients could have been treated either in first or second line. P could also have been associated with other treatments such as carboplatin,^5^, ^10^ which makes comparison with our study difficult in terms of efficacy. Borel et al. showed interesting results rechallenging with platinum +cetuximab chemotherapy regimens for patients progressing after at least 3 months of cetuximab maintenance, with a third of patients having an objective response and median PFS and OS of 6.5 and 11.2 months, respectively,^13^ suggesting that rechallenge with platinum +cetuximab could be an option in this population. However, this study did not only study cetuximab continuation but rechallenge with platinum.

In the P + placebo arm in the BERIL‐1 phase II trial and in our study, median PFS was similar (3.5 months [95% CI 2.2–3.7]). Median OS and response rate were slightly higher: 6.5 months [95% CI 5.3–8.8] and 14%, but patients were selected for phase II (PS 0–1) and PS is known to be a major prognostic factor, as shown in the present study.^14^

The mechanisms of anti‐EGFR resistance are now better understood. Even though EGFR expression does not seem to be a predictive marker in HNSCC,^15^ the presence of an EGFR variant, EGFRvIII, which is a truncated form of EGFR, is present in approximately 40% of cetuximab‐resistant HNSCC.^16^, ^17^ H‐RAS mutations, overexpression of RAS, amphiregulin, or TGF‐β proteins are also escape routes and causes of resistance to EGFR inhibitors.^16^, ^17^, ^18^ Further studies are needed to evaluate the predictive role of these biomarkers.

Immune checkpoints inhibitors are becoming increasingly important in the management of R/M HNSCC. Nivolumab and pembrolizumab, two programmed cell death‐1 (PD‐1) inhibitors, have become new options for patients who have progressed after EXTREME, leading to better OS and ORR than chemotherapy, be it docetaxel, methotrexate, or cetuximab monotherapy.^19^, ^20^ More recently, the Keynote 048 study showed that first‐line immunotherapy with pembrolizumab in monotherapy or in combination with platinum‐based chemotherapy provided better OS than EXTREME regimen.^21^ However, most patients will experience progression after immunotherapy +/‐ chemotherapy and the question of subsequent therapy remains unsolved. Some studies suggest that chemotherapy after immunotherapy is more efficient in lung cancers^22^ and HNSCC,^23^ the latter having objective response rates of up to 30%, that is, well above historical cohorts as a third or fourth line of treatment.

In conclusion, our study suggests that the continuation of cetuximab beyond the first line could provide moderate but significant benefit in OS, PFS, and ORR when combined with paclitaxel. While immunotherapy has become the new standard of care in second line and will probably become the first line, most patients will experience progression, and some will need a subsequent line. PC could fill that gap for selected patients.

## CONFLICT OF INTEREST

C. Le Tourneau has participated in advisory boards of MSD, BMS, Astra Zeneca, Roche, Nanobiotix, Rakuten, GSK, Merck Serono. A. Daste has participated advisory boards of BMS, and Merck Serono. E. Saada‐Bouzid has participated in advisory boards for BMS, Merck Serono, Astra Zeneca. The other authors have no conflict of interest to declare.

## Supporting information

Figure S1

Figure S2

Table S1‐S2
