# Supplementary figures and images for: Cetuximab combined with paclitaxel or paclitaxel alone for patients with recurrent or metastatic head and neck squamous cell carcinoma progressing after EXTREME
Source: Cancer Med. 2021 May 25;10(12):3952–63. doi: 10.1002/cam4.3953 (PMC8209557; doi:10.1002/cam4.3953)

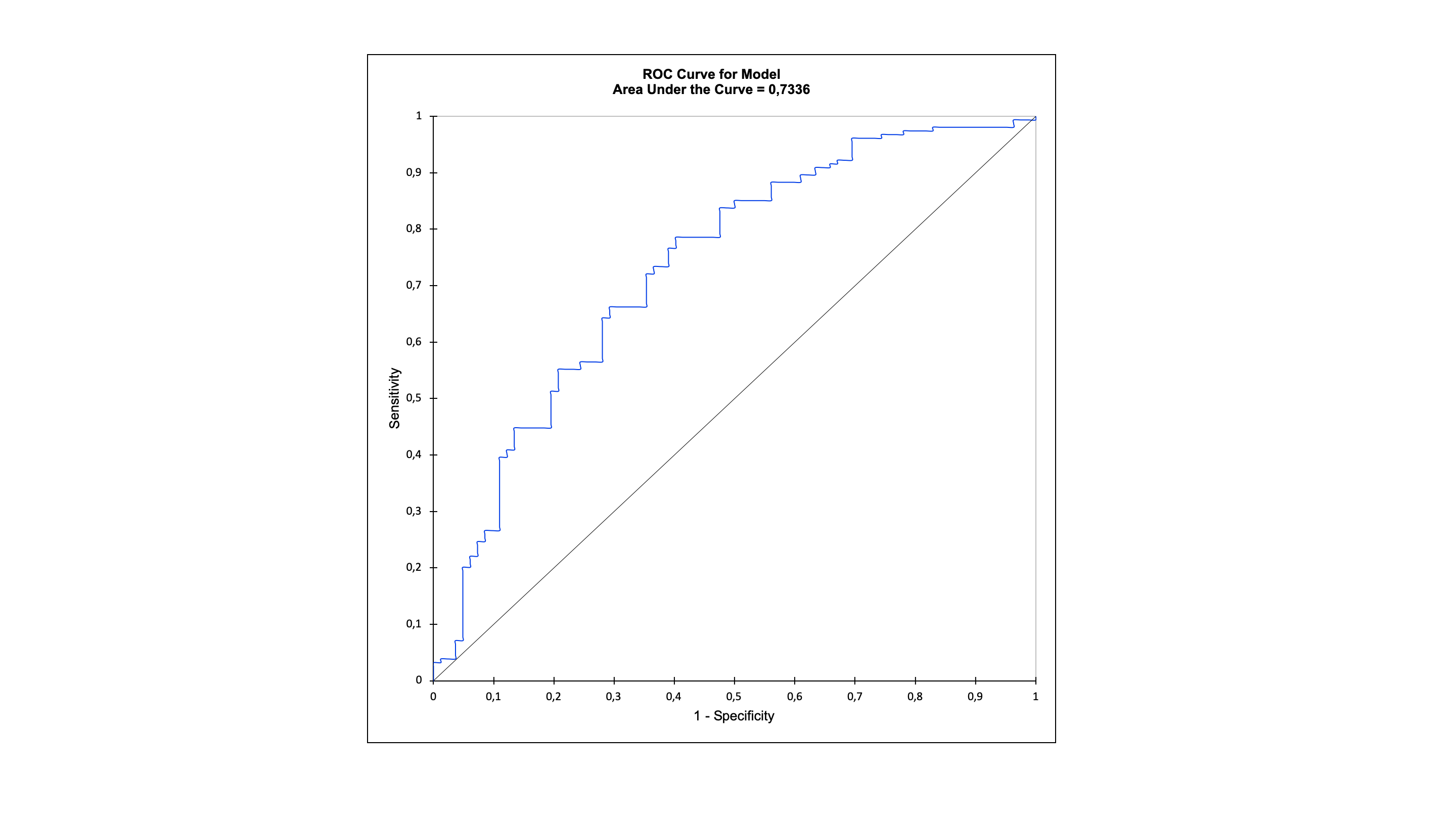

Supplement: Supplementary file 1 — Figure S1 [file CAM4-10-3952-s002.tiff]

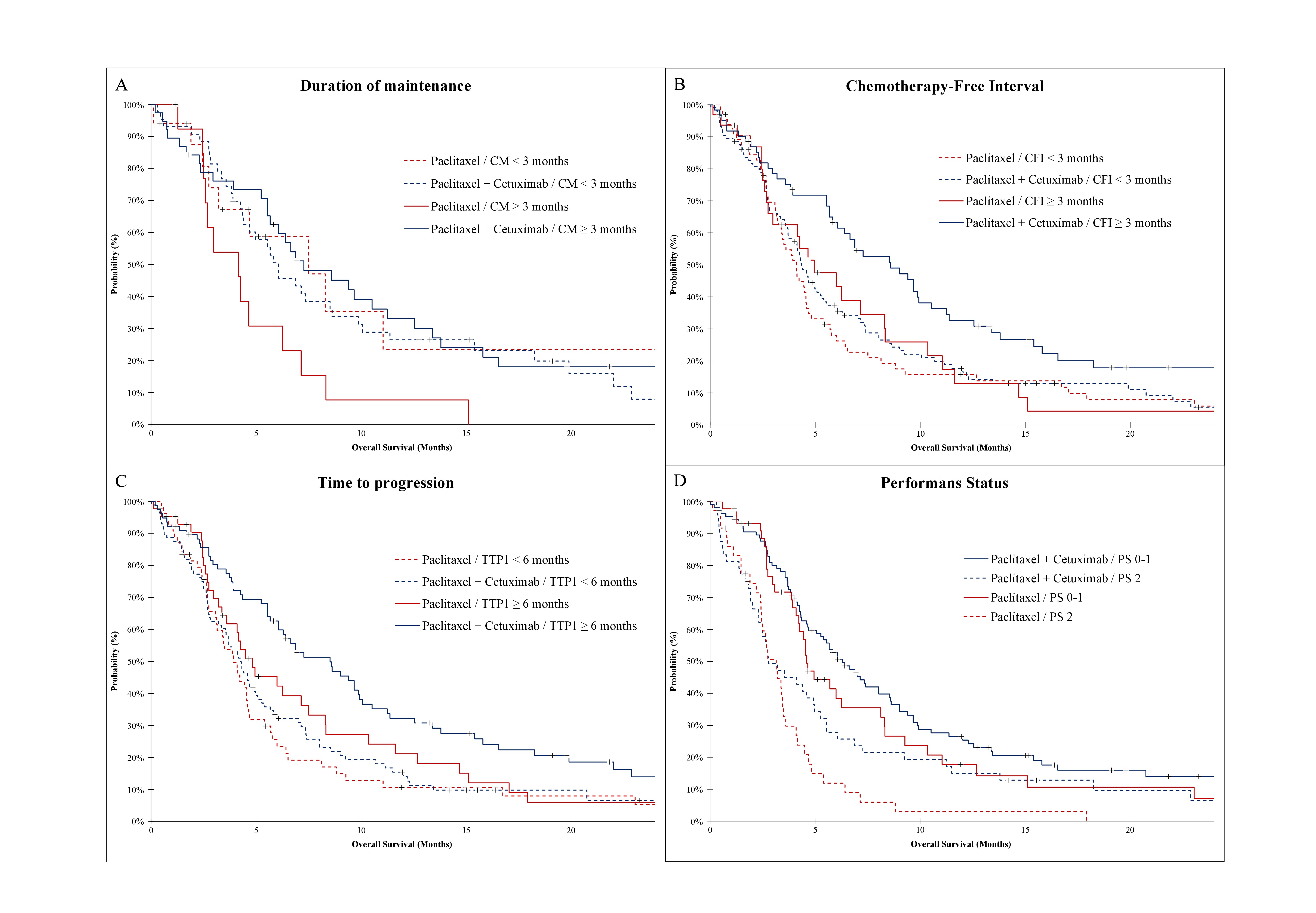

Supplement: Supplementary file 2 — Figure S2 [file CAM4-10-3952-s001.jpg]
