# Supplementary material for: Cetuximab combined with paclitaxel or paclitaxel alone for patients with recurrent or metastatic head and neck squamous cell carcinoma progressing after EXTREME
Source: Cancer Med. 2021 May 25;10(12):3952–63. doi: 10.1002/cam4.3953 (PMC8209557; doi:10.1002/cam4.3953)
Supplement: Supplementary file 3 — Table S1‐S2 [file CAM4-10-3952-s003.docx]

***Supplementary table 1.*** ***Sub-group analysis: Forest Plot for overall survival***

| Factors | | Odds Ratio  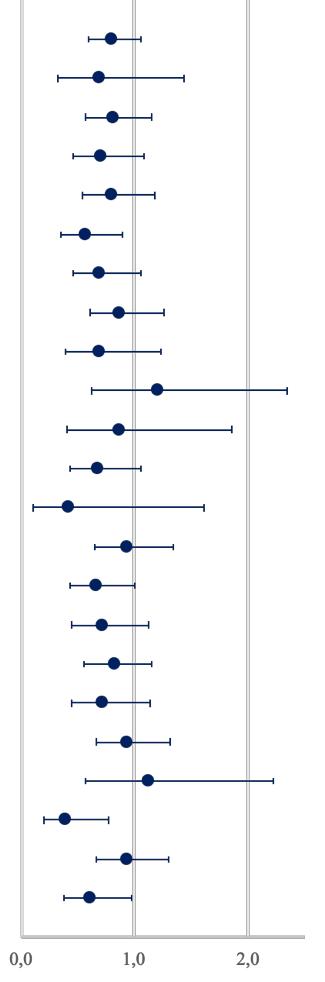[95% CI] |  | *p*-value | *p*-value for  interaction |
| --- | --- | --- | --- | --- | --- |
| **Gender** | Male (n=211) | 0.793 [0.593-1.062] |  | *0.120* | *0.726* |
|  | Female (n=41) | 0.688 [0.329-1.442] |  | *0.322* |  |
| **Age** | < 65 (n=161) | 0.814 [0.576-1.149] |  | *0.242* | *0.607* |
|  | ≥ 65 (n=101) | 0.703 [0.455-1.088] |  | *0.114* |  |
| **Performance  Status** | 0-1 (n=151) | 0.797 [0.542-1.174] |  | *0.251* | *0.260* |
|  | 2 (n=85) | 0.566 [0.359-0.894] |  | ***0.015*** |  |
| **Site of  recurrence** | Locoregional (n=120) | 0.697 [0.462-1.052] |  | *0.085* | *0.422* |
|  | Metastatic (n=141) | 0.873 [0.607-1.255] |  | *0.462* |  |
| **Localization** | Oral Cavity (n=64) | 0.696 [0.393-1.232] |  | *0.213* | *0.763* |
|  | Hypopharynx (n=51) | 1.205 [0.620-2.345] |  | *0.582* | *0.145* |
|  | Larynx (n=39) | 0.863 [0.400-1.859] |  | *0.706* | *0.142* |
|  | Oropharynx (n=102) | 0.679 [0.437-1.055] |  | *0.085* | *0.386* |
|  | Unknown (n=12) | 0.414 [0.107-1.607] |  | *0.202* | *0.360* |
| **TTP1** | < 6 months (n=142) | 0.937 [0.653-1.346] |  | *0.726* | *0.216* |
|  | ≥ 6 months (n=120) | 0.663 [0.439-1.000] |  | ***0.050*** |  |
| **Response  to EXTREME** | Yes (n=99) | 0.713 [0.450-1.130] |  | *0.149* | *0.623* |
|  | No (n=163) | 0.822 [0.588-1.149] |  | *0.251* |  |
| **Cetuximab  maintenance** | Yes (n=112) | 0,713 [0.447-1.137] |  | *0,155* | *0,360* |
|  | No (n=150) | 0,935 [0.662-1.319] |  | *0,700* |  |
| **Duration of maintenance** | < 3 months (n=60) | 1,126 [0.570-2.223] |  | *0,733* | ***0,033*** |
|  | ≥ 3 months (n=52) | 0,397 [0.204-0.774] |  | ***0,007*** |  |
| **Chemotherapy-free interval** | < 3 months (n=169) | 0,932 [0.670-1.296] |  | *0,674* | *0,146* |
|  | ≥ 3 months (n=93) | 0,605 [0.375-0.976] |  | ***0,039*** |  |

***Supplementary table 2. Sub-group analysis: Forest Plot for progression-free survival***

| Factors |  | 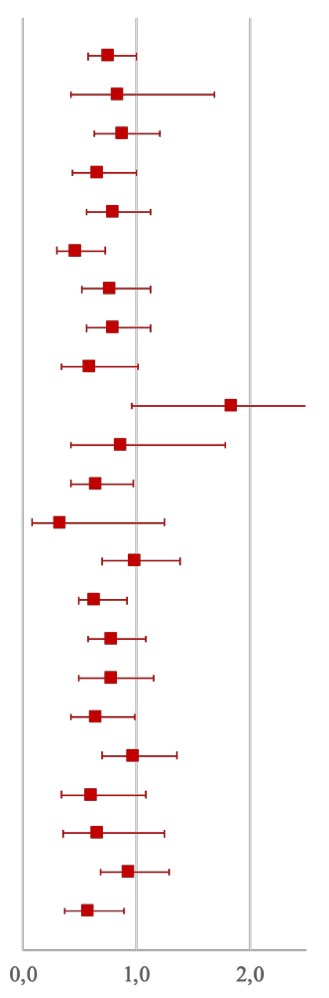Odds Ratio  [95% CI] |  | *p*-value | *p*-value for  interaction |
| --- | --- | --- | --- | --- | --- |
| Gender | Male (n=211) | 0.753 [0,571-0.994] |  | ***0.045*** | *0.772* |
|  | Female (n=41) | 0.841 [0.420-1.686] |  | *0.626* |  |
| Age | < 65 (n=161) | 0.873 [0.631-1.208] |  | *0.414* | *0.293* |
|  | ≥ 65 (n=101) | 0.656 [0.430-1.001] |  | ***0.050*** |  |
| Performance  Status | 0-1 (n=151) | 0.792 [0.555-1.129] |  | *0.197* | *0.069* |
|  | 2 (n=85) | 0.467 [0.298-0.730] |  | ***0.001*** |  |
| Site of  recurrence | Locoregional (n=120) | 0.762 [0.517-1.122] |  | *0.169* | *0.855* |
|  | Metastatic (n=141) | 0.800 [0.566-1.130] |  | *0.205* |  |
| Localization | Oral Cavity (n=64) | 0.587 [0.340-1.013] |  | *0.056* | *0.322* |
|  | Hypopharynx (n=51) | 1.833 [0.959-3.506] |  | *0.067* | ***0.002*** |
|  | Larynx (n=39) | 0.861 [0.417-1.777] |  | *0.685* | *0.767* |
|  | Oropharynx (n=102) | 0.642 [0.426-0.968] |  | ***0.034*** | *0.147* |
|  | Unknown (n=12) | 0.321 [0.083-1.244] |  | *0.100* | *0.194* |
| TTP1 | < 6 months (n=142) | 0.980 [0.692-1.388] |  | *0.911* | *0.880* |
|  | ≥ 6 months (n=120) | 0.623 [0.494-0.915] |  | ***0.016*** |  |
| Response  to EXTREME | Yes (n=99) | 0.785 [0.570-1.083] |  | *0.140* | *0.877* |
|  | No (n=163) | 0.783 [0.492-1.153] |  | *0.192* |  |
| Cetuximab  maintenance | Yes (n=112) | 0,639 [0.416-0.980] |  | ***0,040*** | *0,129* |
|  | No (n=150) | 0,972 [0.698-1.353] |  | *0,866* |  |
| Duration of maintenance | < 3 months (n=60) | 0,603 [0.337-1.076] |  | *0,087* | *0,835* |
|  | ≥ 3 months (n=52) | 0,660 [0.348-1.253] |  | *0,204* |  |
| Chemotherapy-free interval | < 3 months (n=169) | 0,937 [0.683-1.286] |  | *0,687* | *0,074* |
|  | ≥ 3 months (n=93) | 0,570 [0.366-0.888] |  | ***0,013*** |  |
